# HB-EGF Activates the EGFR/HIF-1α Pathway to Induce Proliferation of Arsenic-Transformed Cells and Tumor Growth

**DOI:** 10.3389/fonc.2020.01019

**Published:** 2020-06-30

**Authors:** Lin Wang, Yi-Fan Lu, Chao-Shan Wang, Yun-Xia Xie, Yan-Qiu Zhao, Ying-Chen Qian, Wei-Tao Liu, Min Wang, Bing-Hua Jiang

**Affiliations:** ^1^Department of Pathology, Nanjing Medical University, Nanjing, China; ^2^The Academy of Medical Sciences, Zhengzhou University, Zhengzhou, China; ^3^Department of Internal Medicine, Affiliated Cancer Hospital of Zhengzhou University, Zhengzhou, China; ^4^Department of Pathology, The University of Iowa, Iowa City, IA, United States

**Keywords:** HB-EGF, arsenic, lung cancer, PKM2, HIF-1α

## Abstract

Arsenic was recently identified as a pollutant that is a major cause of lung cancer. Since heparin-binding EGF-like growth factor (HB-EGF) was reported to be a promising therapeutic target for lung cancer, we investigated the role and mechanism of HB-EGF during arsenic-induced carcinogenesis and development of lung cancer. HB-EGF expression were upregulated in As-T cells, lung cancer cell lines, and in most lung cancer tissue samples; and HB-EGF activated the EGFR/p-ERK/HIF-1α pathway and induced VEGF by regulating HIF-1α transcription. HIF-1α transcriptional stimulation by HB-EGF was facilitated by PKM2 and played an important role in HB-EGF's effect on cells. An HB-EGF inhibitor(CRM197, cross-reacting material 197) slowed cell proliferation and inhibited migration of As-T and A549 cells, and inhibited tumor growth. PKM2 also played an important role in the proliferation and migration in As-T cells. The positive staining ratios of EGFR phosphorylation (Y1068) and PKM2 were significantly higher in most cases of lung cancer than in paired normal tumor-adjacent lung tissues; and HB-EGF expression levels strongly correlated with p-EGFR expression levels. Thus, HB-EGF drives arsenic-induced carcinogenesis, tumor growth, and lung cancer development via the EGFR/PKM2/HIF-1α pathway.

## Introduction

Arsenic is a class I carcinogen as defined by the International Center for Research on Cancer (in 1987) and the US Environmental Protection Agency (in the 1990s) (1–3); and arsenic exposure induces various forms of human cancer, including cancers of the lung, skin, and bladder (4). Yet, arsenic has no mutagenic properties, so its carcinogenic mechanism remains unclear. This uncertainty is complicated by the fact that arsenic compounds can inhibit some 200 kinds of enzymes (5, 6), and can regulate DNA methylation, DNA repair, and promote abnormal expression of proto-oncogenes and members of oncogenic or pathogenic pathways. Arsenic-induced epigenetic dysregulation is proposed to contribute to arsenic's toxicity (7), but studies on these effects have largely focused on DNA methylation (8).

Epidermal growth factor receptor [EGFR; a cell-surface tyrosine kinase receptor of the ErbB/HER oncogene family (9)] is among the cancer-related targets of arsenic (10). Reportedly, EGFR overexpression or mutation plays an important role in tumorigenesis in various types of epithelial cancers, including non-small-cell lung cancer (11), breast cancer (12), colorectal cancer (13), and gastric carcinoma (14). Therefore, several anticancer agents target EGFR, either via blocking antibodies or specific inhibitors. For example, EGFR-specific blocking antibodies (such as Erbitux and Vectibix) have been used to treat colorectal cancer (15, 16), while EGFR inhibitors (like Iressa and Canmanna) have been used to treat non-small-cell lung cancer (17). Nevertheless, as with other anti-cancer drugs, most patients develop resistance to these treatments, so prognosis for these cancers remains extremely poor. To improve treatment strategies, the carcinogenic mechanisms of EGFR should be precisely determined (18).

Arsenic exposure in lung cancer cells reportedly upregulates expression of the EGFR ligand, heparin binding-EGF (HB-EGF) and activates EGFR phosphorylation (p-EGFR at Tyr 1173) (5). HB-EGF is a member of the epidermal growth factor (EGF) family ligands, with a sequence similar to EGF, yet HB-EGF induces cellular proliferation and migration more potently than EGF (19). HB-EGF is upregulated in many cancers, including lung cancer; and increasingly, studies have confirmed that, in tumors, HB-EGF acts through binding and overactivating the EGFR pathway, generating signals for proliferation, differentiation, migration, and cell survival (20). Although arsenic exposure was reported to upregulate levels of HB-EGF, little is known about the function and mechanism of HB-EGF in arsenic–induced lung cancer. As such, our work here addressed several key questions: (1) in arsenic-transformed human lung epithelial BEAS-2B cells, lung cancer cells and tissues, how is the expression of HB-EGF affected? (2) What signaling pathways are regulated by HB-EGF in As-T cells? And (3) what is the function of HB-EGF and its downstream signaling pathways, in As-T cells?

## Materials and Methods

### Cell Culture and Reagents

Human bronchial epithelial BEAS-2B (BEAS-2B) and As-T cells were established and cultured in DMEM medium mixed with 10% heat-inactivated fetal bovine serum (FBS), 100 units/mL penicillin G, and 100 μg/mL streptomycin, as previously described (21). Sodium arsenite (NaAsO_2_) and CRM197 were purchased from Sigma Aldrich (St. Louis, MO). EGFR, p-EGFR, PKM2, p-ERK, β-actin antibodies were purchased from Cell Signaling Technology (Danvers, MA, USA). SiHB-EGF, HB-EGF, PKM2 and siPKM2 were purchased from Santa Cruz Biotechnology (Dallas, TX, USA).

### Cell Transfection

Indicated siRNAs or plasmids were transfected into As-T cells using Lipofectamine reagent (Thermo Fisher, USA). Transfection complexes were prepared according to the manufacturer's instructions.

### Protein Extraction and Western Blotting

Cells were washed with ice-cold PBS buffer, scraped from the dishes on ice, and centrifuged at 12,000 rpm, 4°C for 15 min. Cell lysates were prepared using RIPA buffer supplemented with protease inhibitors (Beyotime, Nantong, China). Lysates were assayed by immiunoblotting, as described previously (22), probing with antibodies against EGFR, PKM2, β-actin (Cell Signaling Technology, Danvers, MA, USA), or HIF-1α (BD Biosciences, Bedford, MA).

### Immunohistochemistry

Each lung cancer tissue array (Wuhan Iwill Biological Technology Co. Ltd, Wuhan, China) contained 35 cases of lung cancer and 35 cases of paired, normal, tumor-adjacent lung tissue. Arrays were deparaffinized, hydrated, pretreated for epitope unmasking, incubated with hydrogen peroxide, blocked with 10% goat serum (according to the manufacturer's instructions), and then arrays were incubated with the indicated antibodies at 4°C overnight. After washing, arrays were treated as previously reported (23).

### Real-Time RT-PCR

Total cellular RNAs were extracted using Trizol reagent according to the manufacturer's instructions. Aliquots of total RNAs (1 μg) were used as templates to synthesize the first-strand cDNAs using reverse transcriptase. PCR primer pairs were as follows: GAPDH forward, 5′-CCACCCATGGCAAATTCCATGGC-3′;

GAPDH reverse, 5′-TCTAGACGGCAGGTCAGGTCCACC-3′;

HB-EGF forward, 5′-ATCGTGGGGCTTCTCATGTTT-3′;

HB-EGF reverse, 5′-TTAGTCATGCCCAACTTCACTTT-3′;

VEGF forward, 5′-TCGGGCCTCCGAAACCATGA-3′;

VEGF reverse, 5′-CCTGGTGAGAGATCTGGTTC-3′.

Real-time PCR was performed using the above primers and SYBR Green Mastermix (Vazyme Biotech Co., Ltd, Nanjing, China). Reactions were analyzed on an ABI 7900 real-time PCR machine using the following cycle conditions: 95°C for 10 min, followed by 40 cycles at 95°C for 15 s and 60°C for 1 min. Results were presented as the ratio of HB-EGF or VEGF to GAPDH and normalized to the control group.

### Construction of Plasmids

The HIF-1α promoter, containing PKM2 binding sites, was amplified from human genomic DNA via PCR. Primers contained KpnI and HindIII restriction enzyme cutting sites:

Forward primer, 5′-AGCTGGTACCCCTGTGTACAAGCTCACG-3′; reverse primer, 5′-AGCTAAGCTTCATGGTGAATCGGTCCC-3′. The PCR product was inserted into the PGL3-Basic plasmid vector and verified by DNA sequencing. The VEGF promoter reporter pMAP11wt, containing HIF-1α binding sites, was inserted into the pGL2 basic luciferase vector, as described previously. The plasmid encoding human HIF-1α was inserted into the pCEP4 vector, as described previously (21, 24).

### Dual-Luciferase Assay

Cells were washed once with PBS buffer and lysed with Reporter Lysis Buffer from Promega (Madison, WI, USA). Luciferase (Luc) activity was measured; and the relative Luc activity was calculated as the Luc activities ratio of the HIF-1α reporter to PGL4.74, and then normalized to that of the control.

### Cell Proliferation Assay

Cell proliferation was assayed using the CCK8 kit (Dojindo, Kumamoto, Japan), according to the manufacturer's instructions, at the indicated time points. One thousand cells per well were seeded and cultured in 96-well plates. All results were obtained from three separate experiments with three replicates per experiment.

### Cell Invasion Assay

A cell-invasion assay was performed using trans-well cell culture inserts (Becton Dickinson) in accordance with the manufacturer's instructions. 5 × 10^4^ cells were seeded per well, in the upper well of the invasion chamber in DMEM without serum. The wells' lower chambers contained DMEM supplemented with 10% FBS to stimulate cell invasion. After a 14-h incubation, non-invading cells were removed from the top well with a cotton swab, while the bottom cells were stained with a crystal violet solution and photographed in three independent fields for each well. Transfected cells were maintained for 48 h and allowed to migrate for an additional 24 h. Passaged cells were stained with a crystal violet solution.

### Animal Experiments

Male BALB/c nude mice (4 weeks old) were purchased from Beijing Vital River Laboratory Animal Technology Co., Ltd (Beijing, China). Animal handling and experimental procedures were performed according to the guide for the Care and Use of Laboratory Animals in Nanjing Medical University. As-T cells were injected subcutaneously into both flanks of nude mice (5 × 10^6^ cells in 100 μL). Tumor sizes were measured using Vernier caliper every 2 days when the tumors were visible and tumor volume was calculated according to the formula: Volume = 0.5 × Length × Width^2^ (25). Twenty-four days after implantation, mice were sacrificed and tumors were dissected.

### Statistical Analysis

All experiments were performed three times and data were analyzed by Microsoft Excel 2016, with the exception of the correlation of HB-EGF with p-EGFR, which was analyzed by GraphPad Prism 7.0. Statistical evaluation of data was performed using the *t*-test. *P* < 0.05 was considered to be statistically significant.

## Results

### HB-EGF Is Upregulated in Arsenic-Transformed Lung Epithelial Cells, Lung Cancer Cells, and Cancer Tissues

**Figure 1:**
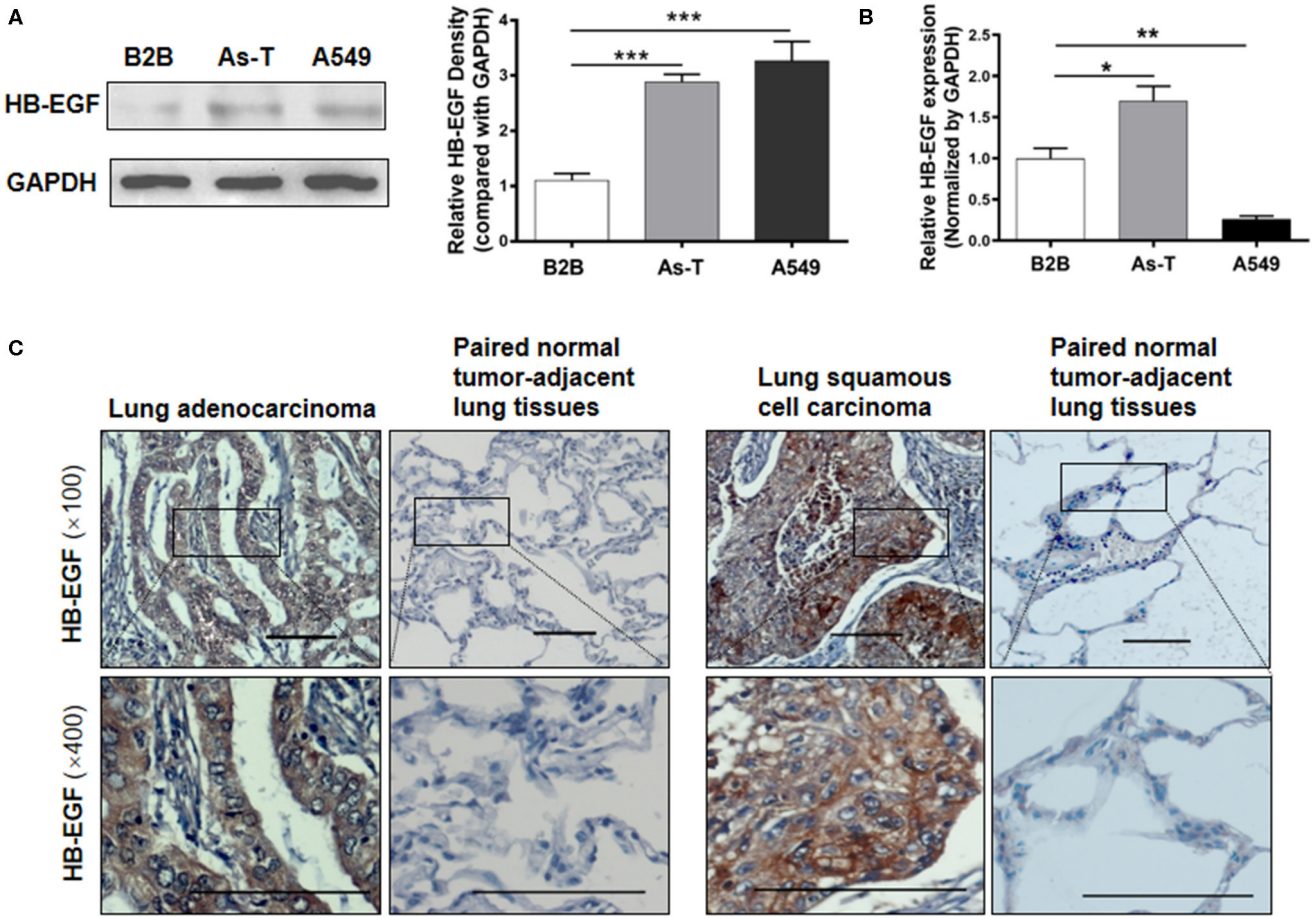
HB-EGF is upregulated in arsenic-transformed cells and lung cancer cells or tissues. **(A)** Protein levels of HB-EGF in B2B, As-T, and A549 cells were determined by Western blotting, with β-actin used as loading control. The relative amount of HB-EGF was analyzed by Tanon Western Blot software (Tanon Science & Technology Co., Ltd, Shanghai China). As-T, arsenic-transformed human lung epithelial BEAS-2B; B2B, BEAS-2B. **(B)** mRNA levels of HB-EGF in B2B, As-T, and A549 cells were analyzed by RT-qPCR, with GAPDH was used as a loading control. **(C)** Representative immunohistochemical-staining images of HB-EGF in lung tumors and matched adjacent tissues (×100 and ×400 magnification; scale bar: 100 μm). The data are presented as the mean±SD. *p < 0.05, **p < 0.01, ***p < 0.001.

Overexpression of HB-EGF is reported in many cancers, including lung cancer so we evaluated HB-EGF protein and mRNA expression levels (by western blotting and qPCR) in arsenic transformed cells and the lung cancer cell line, A549. As we predicted, As-T cells and A549 expressed more HB-EGF protein than in B2B cells (Figure 1A); and interestingly, HB-EGF mRNA expression was highest in As-T cells and lowest in A549 cells (Figure 1B). Similarly, when HB-EGF expression was compared by immunohistochemical staining in 35 pairs of lung cancer tissue from lung squamous cell carcinoma and adenocarcinoma and matched adjacent normal lung tissues, HB-EGF was comparatively overexpressed in most cases (Figure 1C and Table 1).

**Table 1:** Expression of HB-EGF in lung cancer and paired normal tumor-adjacent lung tissues.

				**−**	**+**	**++**	**+++**
**Lung cancer (*****n*** **= 35)**			HB-EGF	9	6	5	15
Well-differentiated lung carcinoma (*n* = 11)	Moderately differentiated lung carcinoma (*n* = 13)	Poorly differentiated carcinoma (*n* = 11)					
Paired normal tumor-adjacent lung tissues (*n* = 35)			HB-EGF	29	3	1	2

*Statistical data of HB-EGF expression in lung tumor samples and matched, adjacent tissues. Statistical data of immunohistochemical staining of HB-EGF in 35 lung-tumor samples and matched adjacent tissues. “–” indicates negative staining; “+,” “++,” “+++” indicate increments of positive staining*.

### HB-EGF Activates EGFR in Arsenic-Transformed Cells

**Figure 2:**
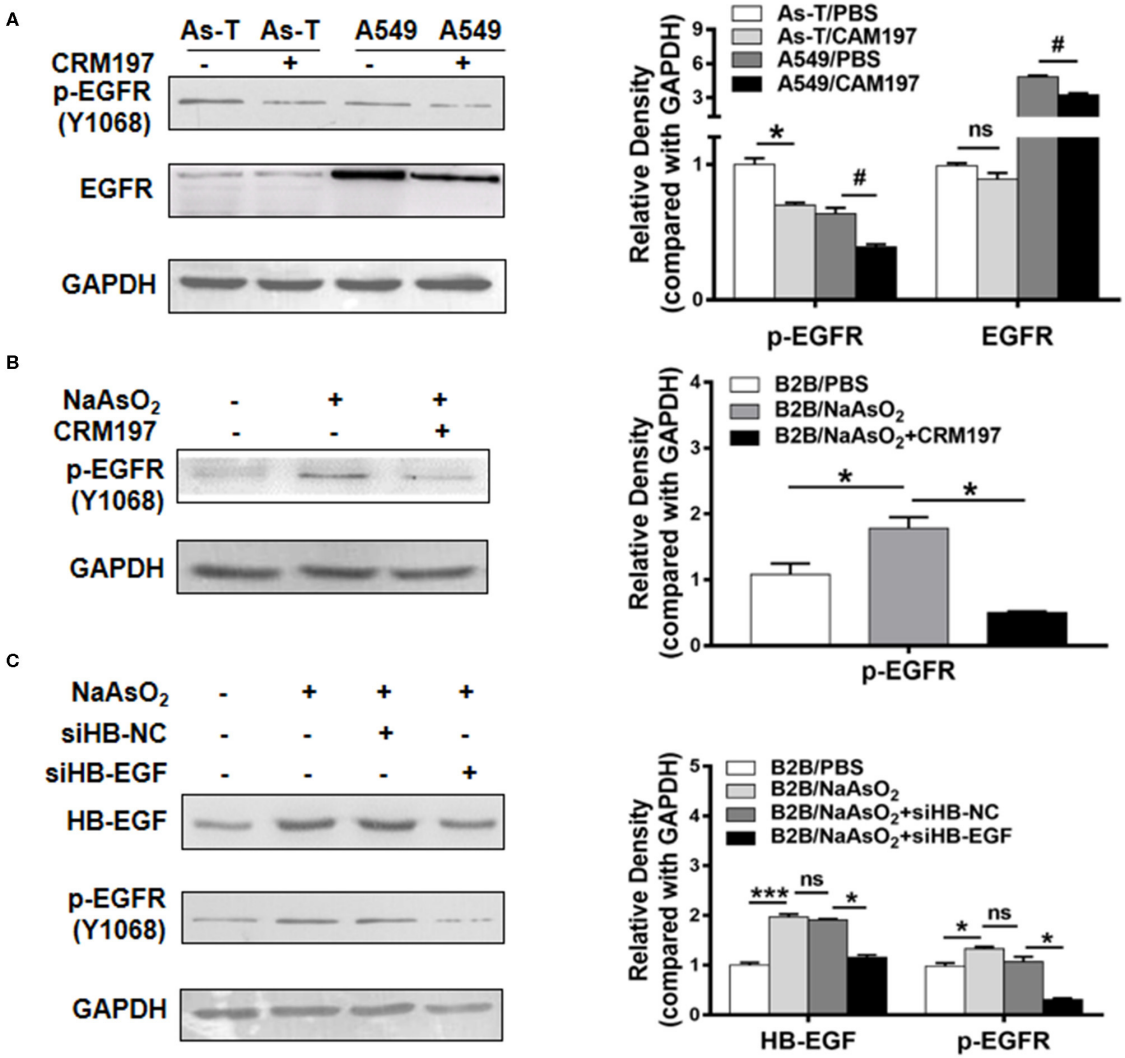
HB-EGF activates EGFR in arsenic transformed cells. **(A)** As-T cells and A549 cells were treated with 10 μg/mL CAM197 (an HB-EGF inhibitor). After 48 h, p-EGFR(Y1068) and EGFR expression were analyzed via Western blotting. CAM197, cross-reacting material 197; p-EGFR (Y1068), activated EGFR at phospho-Tyr1068. *p < 0.05, compared with As-T/PBS. ^#^*p* < 0.05, compared with A549/PBS. **(B)** B2B cells were treated with or without 1 μM NaAsO_2_ and 10 μg/mL CRM197, as indicated for 48 h; and levels of p-EGFR protein were determined via Western blotting. *p < 0.05, compared with B2B/PBS. **(C)** B2B cells were transfected with siHB-NC and siHB-EGF as per the manufacturer's instructions. Twelve hours after transfection, As-T cells were treated with or without NaAsO_2_, as indicated for 36 h, and expression of HB-EGF and p-EGFR was analyzed by Western blotting. The data are presented as the mean±SD. *^,#^*p* < 0.05, ***p < 0.001.

Reportedly, in lung cancer cells, arsenic exposure upregulates the levels of heparin binding-EGF (an EGFR ligand) and stimulates an activating phosphorylation of EGFR (p-EGFR at Tyr 1173). To test whether arsenic activates EGFR through HB-EGF, As-T and A549 cells were first treated with 10 μg/mL of CRM197 (an HB-EGF inhibitor; as indicated in Figure 2A) for 48 h, and p-EGFR and EGFR protein levels were evaluated by Western blotting. Under these conditions, CRM197 inhibited p-EGFR protein levels, in both As-T and A549 cells, but it did not affect EGFR protein level in As-T cells. When B2B cells were treated for 48 h with 1 μM NaAsO_2_ and 10 μg/mL CRM197, as indicated in Figure 2B, the arsenic-induced increases in p-EGFR expression were reversed, suggesting arsenic activated p-EGFR via the HB-EGF ligand. Confirmatory results (shown in Figure 2C) showed transfecting an siHB-EGF similarly inhibited p-EGFR protein levels.

### HB-EGF Induces ERK Activation and Increases HIF-1α Expression

**Figure 3:**
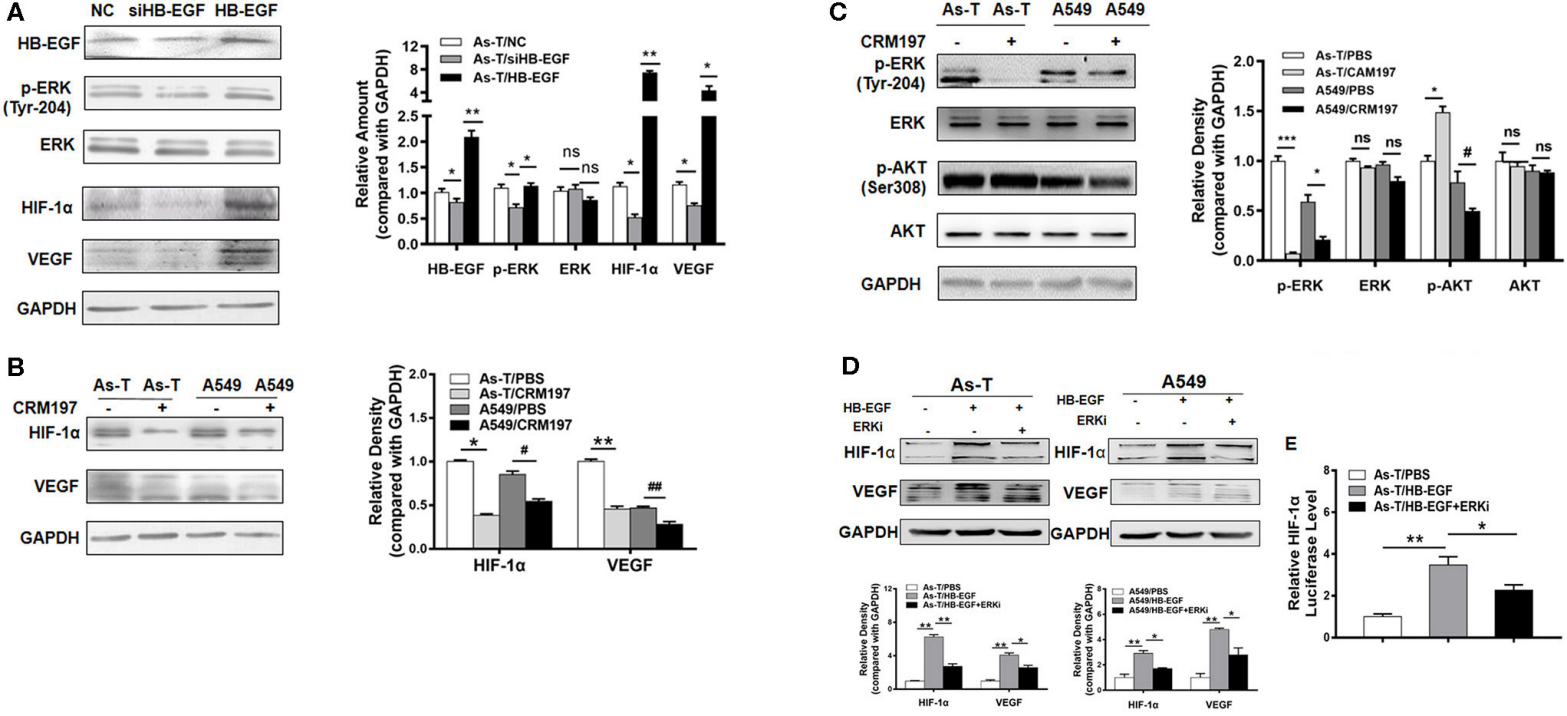
HB-EGF upregulates p-ERK/ HIF-1α pathway in Arsenic transformed cells. **(A)** As-T cells were transfected with siHB-EGF or treated with HB-EGF and, after 48 h, HB-EGF, p-ERK, HIF-1α, and VEGF were tracked, as above. **(B)** As-T and A549 cells were treated with or without CAM197 as indicated for 48 h, and p-ERK, p-AKT and **(C)** HIF-1α and VEGF were tracked, as above. GAPDH was used as loading control. **(D)** As-T and A549 cells were treated with HB-EGF and an ERK inhibitor, as indicated for 48 h; and HIF-1α and VEGF were tracked by Western blotting. **(E)** As-T cells were transfected with a HIF-1α promoter reporter (containing PKM2 binding sites). After 12 h, cells were exposed to HB-EGF and an ERK inhibitor, for 36 h, and luciferase (Luc) activities were determined. The data are presented as the mean±SD. *^,#^*p* < 0.05, **^,##^*p* < 0.01, ***p < 0.001.

To study whether HB-EGF upregulated EGFR downstream of the pro-oncogenic signaling pathways, MAPK/ERK and PI3K/AKT (26), As-T cells were transfected with our siHB-EGF or a vector that overexpressed HB-EGF. Cells were then assessed for expression of p-ERK, HIF-1α, and VEGF by Western blotting (Figure 3A). Since ERK/HIF-1α/VEGF signaling reportedly regulates tumorigenesis in several cancers (24), we hypothesized HIF-1α might be suppressed by CRM197. To test this hypothesis, HIF-1α and VEGF expression were evaluated by Western blotting in As-T and A549 cells exposed to CRM197 for 48 h. As shown in Figure 3B, CRM197 inhibited HIF-1α and VEGF expression significantly in both As-T and A549 cell types. When As-T cells were treated with 10 μg/mL CRM197, p-ERK expression was inhibited, but not p-AKT (Figure 3C). These results were confirmed in studies showing HB-EGF-induced ERK activation stimulated expression of HIF-1α and VEGF. Furthermore, to confirm that HB-EGF upregulated expression of HIF-1α and VEGF via ERK, As-T cells were treated with HB-EGF and the inhibitor of ERK (ERKi; Figure 3D) for 48 h. Western blotting results of HIF-1α and VEGF showed that the effect of HB-EGF upregulated HIF-1a and VEGF can be reversed by ERK inhibitor, suggesting HB-EGF upregulated HIF-1α and VEGF via ERK (Figure 3D).

To test whether HB-EGF increases HIF-1α transcriptional activation, an HIF-1α promoter driving a luciferase reporter was transfected into As-T cells, which were cultured overnight and then treated with HB-EGF or the ERK inhibitor for 24 h. Comparing luciferase activities showed HB-EGF treatment significantly increased HIF-1α reporter activity in As-T cells, while the ERK inhibitor completely restored HB-EGF's effect on HIF-1α at the transcriptional level (Figure 3E). These results demonstrate that, in As-T cells, HB-EGF increased HIF-1α transcription via ERK.

### PKM2 Upregulates HIF-1α at the Transcriptional Level via ERK and Stimulates the Proliferation and Migration of Arsenic-Transformed Cells and A549 Cells

Embryonic pyruvate kinase M2 (PKM2) is one of four isoforms of pyruvate kinase, an enzyme that catalyzes the conversion of phosphoenolpyruvate and ADP to pyruvate and ATP during glycolysis (27). PKM2 is highly expressed in human cancer and has a central role in the metabolic reprogramming of cancer cells as well as participating in cell-cycle progression and gene transcription (28). EGFR activation induces translocation of PKM2 into the nucleus where, in hypoxic human cancer cells, PKM2 physically interacts with and promotes transactivation of HIF-1α (21, 25, 29, 30).

**Figure 4:**
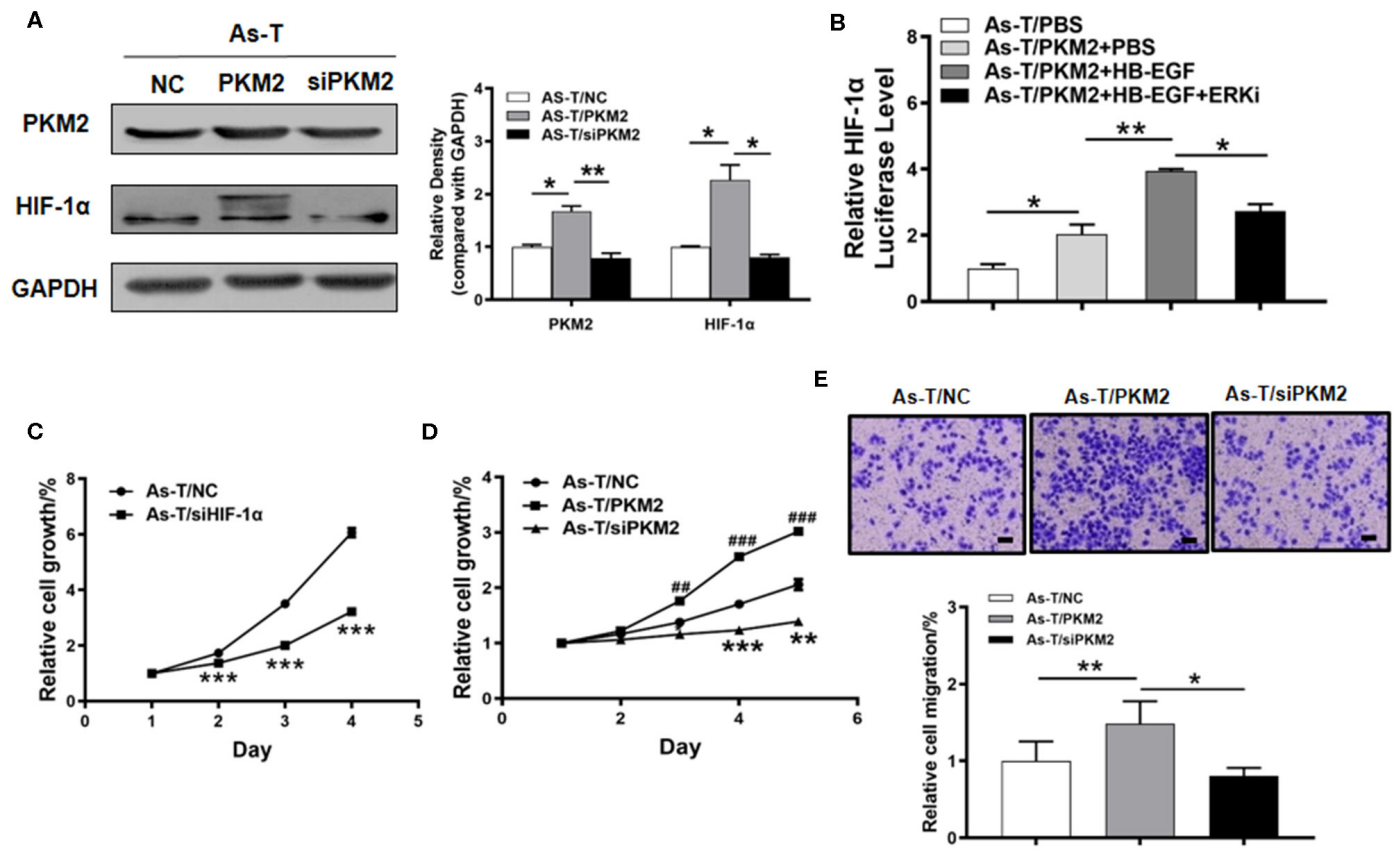
PKM2 upregulates HIF-1α via Erk and is important in regulating proliferation of arsenic-transformed cells**. (A)** Arsenic transformed cells were transfected with vectors expressing NC, PKM2 or siPKM2, and 60 h later, PKM2 and HIF-1α expression was tracked using Western blotting. **(B)** As-T cells were co-transfected with an HIF-1α promoter reporter (containing PKM2 binding sites) and PKM2. After 6 h, the cells were cultured with an ERK1/2 inhibitor or HB-EGF, as indicated, and luciferase (Luc) activities were determined. **(C)** The CCK-8 assay monitored proliferation of As-T cells transfected with an HIF-1α expression vector or a control, according to the manufacturer's instruction. Proliferation rates were determined daily for 4 days after seeding. **(D)** As-T cells were analyzed as in **(C)**, after transfection with PKM2, ashPKM2 plasmid, or a control. **(E)** PKM2's effect on trans-well migration of As-T cells. Passaged cells were stained with a crystal violet solution and counted. Data are presented as the mean±SD. *p < 0.05, **^,##^*p* < 0.01, ***^,###^*p* < 0.001.

To determine whether PKM2 regulated HIF-1α in As-T cells, they were transfected with or siPKM2 RNA or a vector expressing PKM2. As shown in Figure 4A, PKM2 overexpression significantly increased HIF-1α expression; and, conversely, siPKM2 inhibited HIF-1α expression. Since ERK inhibition was reported to block PKM2 translocation into the nucleus, we assumed that the ERK inhibitor reversed the HIF-1α transcriptional activation. To test this assumption, As-T cells were transfected with a HIF-1α promoter reporter plasmid and PKM2. After overnight culture, the cells were treated for 24 h with HB-EGF or the ERK inhibitor, and luciferase activity was measured. Consistent with previous results, PKM2 increased HIF-1α transcriptional activation and HB-EGF upregulated PKM2's stimulatory effect on HIF-1α transcriptional activation; moreover, the ERK inhibitor completely restored the PKM2-stimulating, HIF-1α transcriptional activation (Figure 4B).

To study the biological function of HIF-1α and PKM2 in arsenic-induced lung cancer, As-T cells were transfected with siHIF-1α or siPKM2, and a vector expressing PKM2. After 24 h, forced expression of siHIF-1α inhibited the proliferation rate of As-T cells as compared to the control (Figure 4C). After 36 h, PKM2 expression significantly promoted As-T cell growth (Figure 4D), while on the contrary, siPKM2 significantly inhibited their proliferation and migration (Figures 4D,E). These results suggest upregulation of HIF-1α by PKM2 via the ERK pathway plays important role in As-T cells.

### siHB-EGF Inhibits VEGF at the Transcriptional Level in Arsenic-Transformed Cells by Depressing HIF-1α

**Figure 5:**
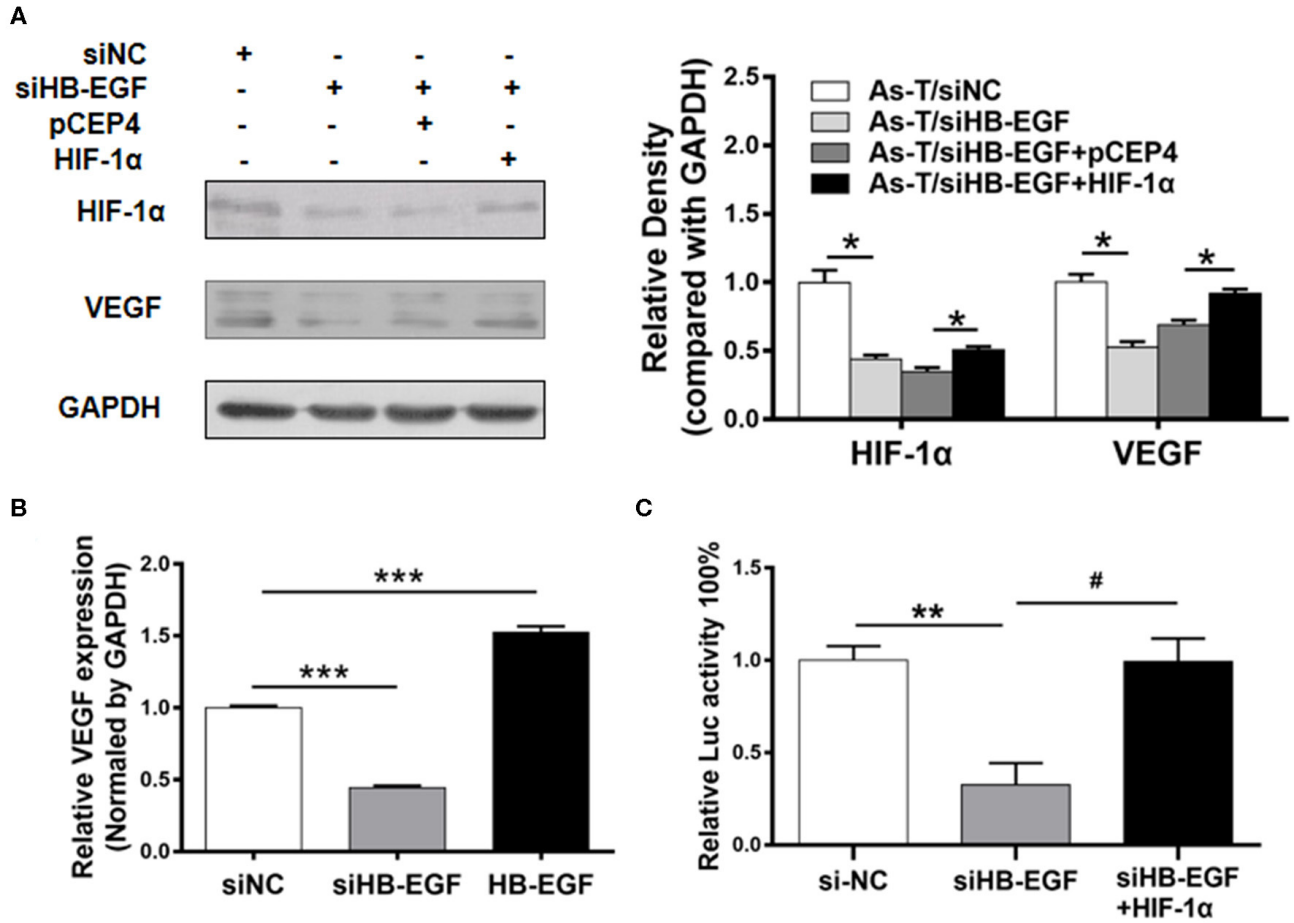
siHB-EGF inhibits VEGF at the transcriptional level, in arsenic transformed cells, by inhibiting HIF-1α. **(A)** As-T cells were transfected with or without siNC, siHB-EGF, and vectors expressing pCEP4 and HIF-1α, as indicated. After 48 h, expression of HIF-1α and VEGF was analyzed, as above. GAPDH was used as a loading control. **(B)** As-T cells were transfected with siHB-EGF, and vectors expressing NC or HB-EGF. Expression of VEGF mRNA was determined by RT-qPCR 48 h after transfection. Expression of VEGF mRNA was normalized to GAPDH expression. Data are presented as the mean±SD. *** Indicates a significant difference compared to the NC control (*P* < 0.001). **(C)** As-T cells were co-transfected with pMAP11wt reporter plasmid (a VEGF promoter reporter containing HIF-1α binding sites), and siHB-EGF, or a vector expressing HIF-1α for 48 h. Relative Luc activity was determined. The data are presented as the mean±SD. *^,#^*p* < 0.05, **p < 0.01, ***p < 0.001.

VEGF is a key regulator of several aspects of cancer that are linked to poor patient prognosis, including tumor growth, angiogenesis, metastasis, and progression. Also, various approved antitumor modalities target pathways downstream of VEGF and EGFR (31). VEGF expression is mainly regulated at the transcriptional level by hypoxia-inducible factor 1α (HIF-1α), which binds the VEGF promoter hypoxia response element (HRE) (24). To confirm HB-EGF increased VEGF expression through HIF-1α, As-T cells were transfected with siHB-EGF or a vector expressing HIF-1α for 48 h, and HIF-1α, VEGF and GAPDH expression was tracked by Western blotting. The results showed siHB-EGF decreased HIF-1α and VEGF protein levels, and that these could be restored by overexpressing HIF-1α. When levels of VEGF and GAPDH mRNA in As-T cells were determined by real-time RT-PCR, we found siHB-EGF suppressed VEGF mRNA levels. In parallel, HB-EGF increased VEGF (Figures 5A,B).

To determine whether overexpression of HIF-1α was sufficient to restore the ability of siHB-EGF to inhibit the VEGF transcription, As -T cells were co-transfected with siHB-EGF, HIF-1α, and VEGF reporter plasmids. These studies indicated when suppression of VEGF transcriptional activation by siHB-EGF could be reversed by HIF-1α overexpression, demonstrating that siHB-EGF inhibited VEGF expression by suppressing HIF-1α expression (32) (Figure 5C).

### Inhibitors of HB-EGF Inhibit Proliferation and Migration of As-T and A549 Cells

**Figure 6:**
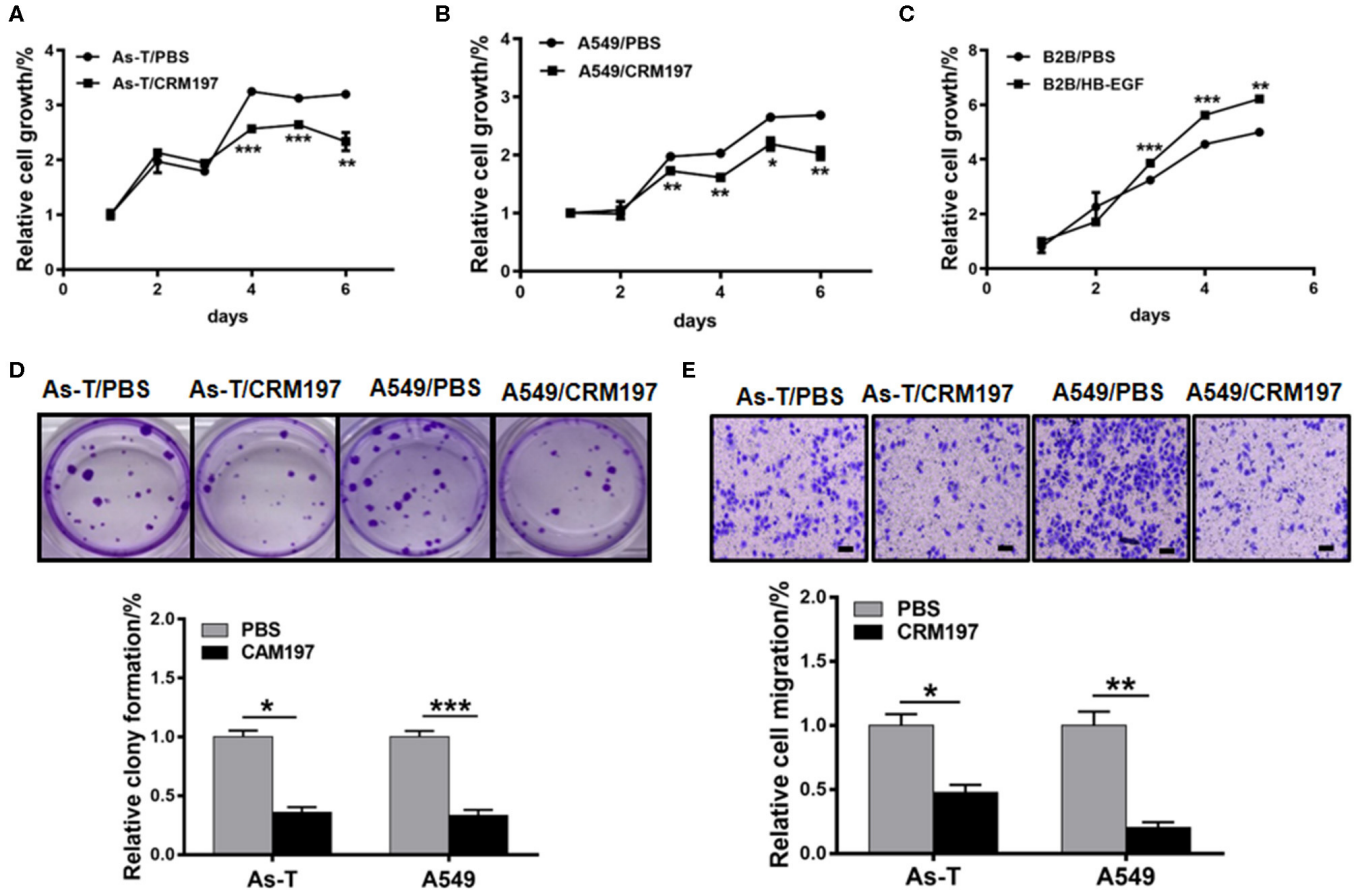
An HB-EGF inhibitor impedes proliferation, migration, and tumor growth**. (A)** As-T cells or A549 cells **(B)** were treated with 10 μg/mL CRM197, and relative cell growth was determined by the CCK8 assay. **(C)** B2B cells were treated with HB-EGF, and relative cell growth was determined by the CCK8 assay. **(D)** A colony-formation assay was performed using As-T and A549 cells treated with or without 10 μg/mL CAM197 for 14 days. The upper panel shows representative colonies. The lower data were represented as the mean±SD, from five replicates of each treatment. **(E)** Migration of As-T and A549 cells treated with CAM197, using a trans-well assay. Representative migration is shown in the upper panel. The data are presented as the mean±SD. *p < 0.05, **p < 0.01, ***p < 0.001.

To study the function of HB-EGF in As-T and A549 cells, the cells were treated with 10 μg/mL CRM197. CRM197 inhibited As-T cell proliferation after 48 h of treatment, compared to controls (Figure 6A). Similarly, A549 cell proliferation was significantly inhibited after treatment with CRM197 for 36 h (Figure 6B). Conversely, HB-EGF promoted B2B cell growth, after 36 h of treatment exposure (Figure 6C). A colony-formation assay validated the anti-tumor effects of CRM197 in As-T and A549 cells (Figure 6D); and a transwell migration assay (Figure 6E) showed migration of As-T and A549 cells decreased by more than 2-fold after exposure to CRM197.

### A Specific Inhibitor of HB-EGF (CRM197) Blocks Tumor Growth *in vivo*

**Figure 7:**
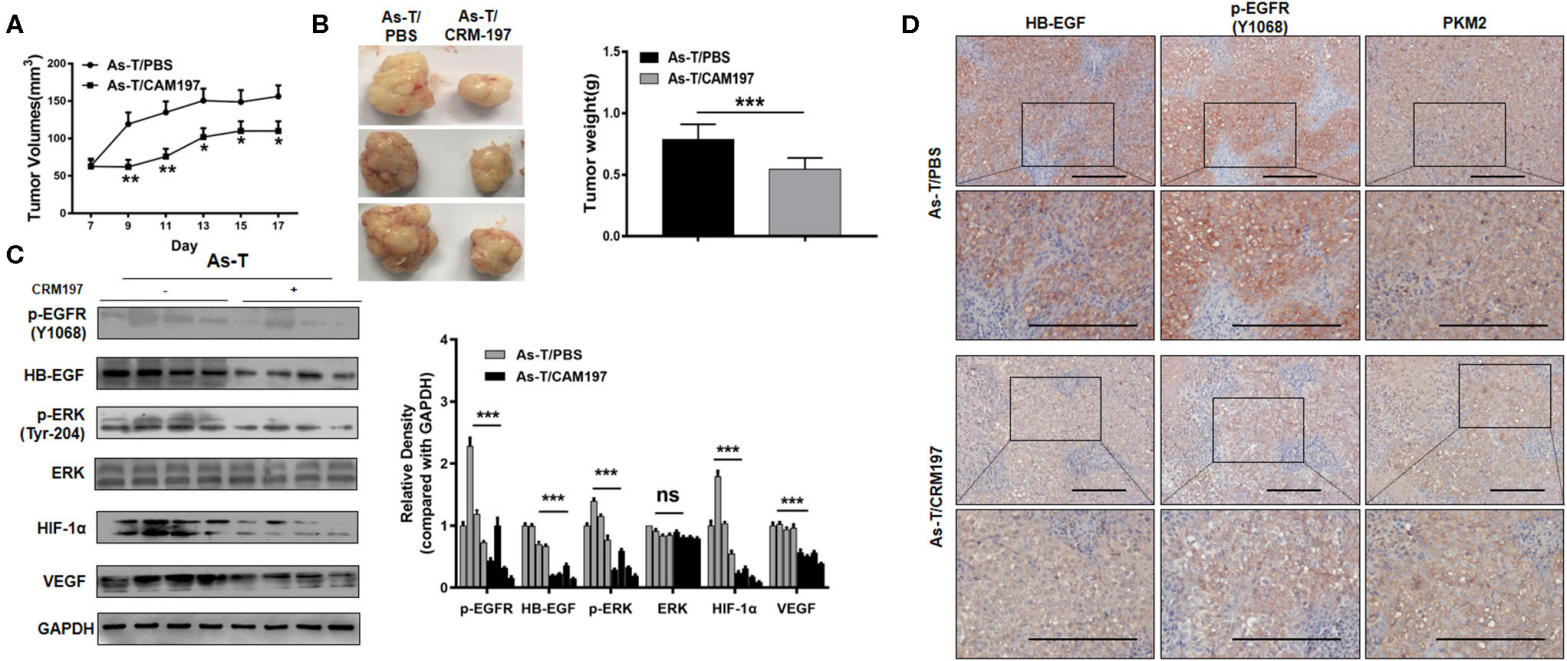
CRM197 inhibits tumor growth *in vivo***. (A)** Nu/nu mice (5 to 6 weeks old) were subcutaneously injected with 5 × 10^6^ As-T cells. Seven days later, CRM197 or PBS was injected into any visible tumors every day. Tumor size was measured daily, as indicated. Tumor volumes were calculated using the formula 0.5×length×width. **(B)** Tumors were resected and weighed after 10 days of injection treatment. Data are presented as the mean±SD. **indicates a significant difference at *P* < 0.01. **(C)** The levels of p-EGFR protein and its downstream-signaling-pathway proteins in tumors, as determined by Western blotting. Representative western blotting images are shown. **(D)** Immunohistochemistry tracking the expression of HB-EGF, p-EGFR, and PKM2, in PBS/CRM197 mouse tumor tissues. The data are presented as the mean±SD. *p < 0.05, **p < 0.01, ***p < 0.001.

To study the effect of CRM197 on tumor growth *in vivo*, As-T cells were injected into nude mice and, after 7 days, the mice were treated with CRM197 or PBSonce every day for 10 days. As shown in Figures 7A,B, CRM197 treatment inhibited tumor growth; and Western blotting showed CRM197 inhibited the expression of p-EGFR, p-ERK, HIF-1α, and VEGF *in vivo* (Figure 7C), consistent with our *in vitro* results. Immunohistochemistry verified that CRM197 inhibited HB-EGF, p-EGFR, and PKM2 expression in mouse tumor tissues (Figure 7D).

### Higher Levels of HB-EGF and PKM2 Are Associated With Cancer Development

**Figure 8:**
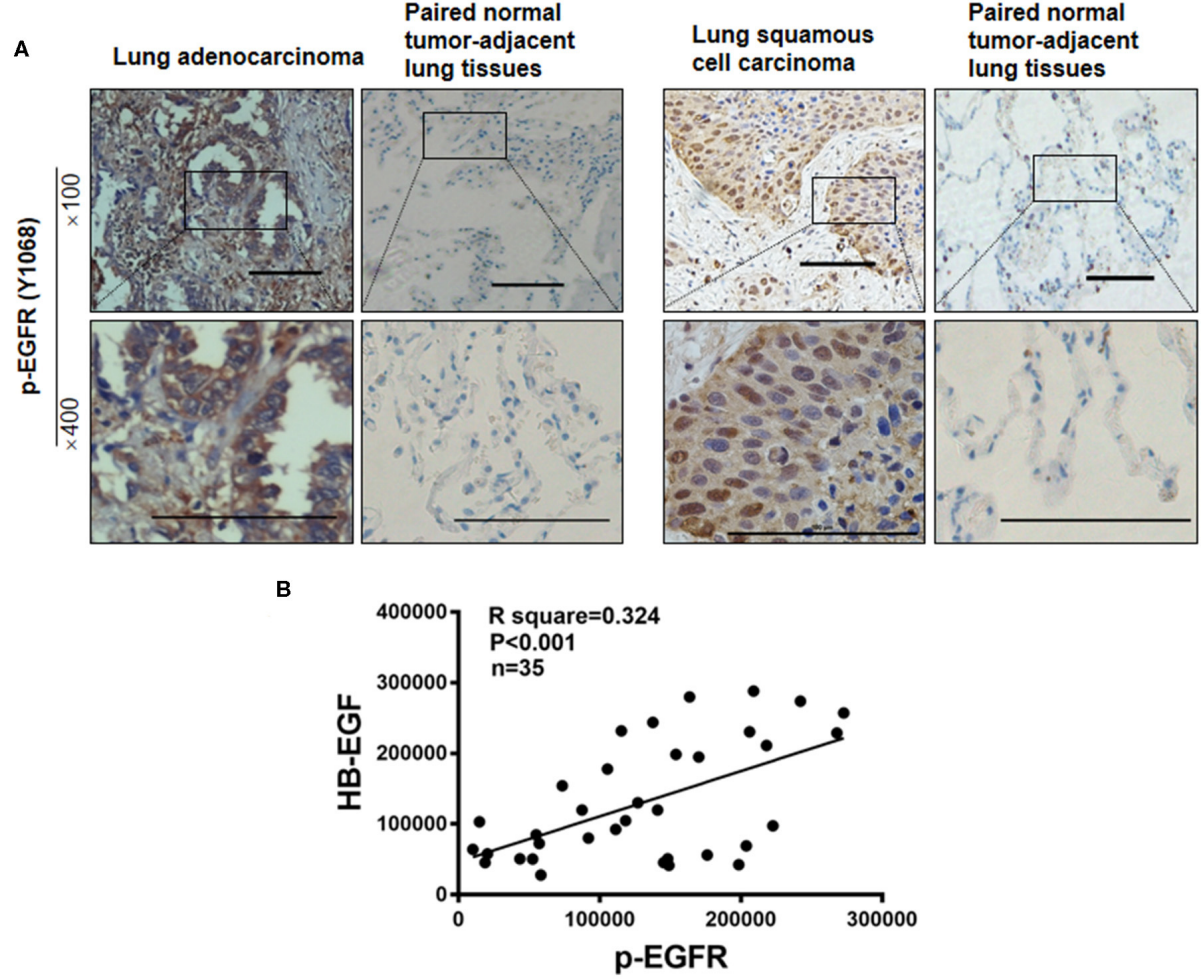
p-EGFR are overexpressed in most cases of lung cancer. **(A)** p-EGFR was immunohistochemically stained in 35 lung cancer tumor samples and paired, normal, tumor-adjacent lung tissue, respectively. Representative immunohistochemical images are shown. **(B)** IHC scores as measured by Image Pro Plus 6.0. Pearson Correlation analysis shows IHC scores of 35 pairs of lung cancer tissues between HB-EGF and p-EGFR. R square = 0.3249, *r* = 0.57, *n* = 35.

To test whether levels of HB-EGF, p-EGFR, and PKM2 correlate in clinical lung cancer tissues, their expression levels were tracked in 35 pairs of lung cancer tissues, by immunohistochemical staining. HB-EGF, p-EGFR, and PKM2 were overexpressed in most cases of lung cancer tissues compared with their matched adjacent normal tissues (representative images are shown in Figures 1C, 8A; Tables 1, 2). Pearson Correlation analysis identified significant positive correlations between HB-EGF and p-EGFR expression (Figure 8B), but HB-EGF and p-EGFR expression did not correlate with that of PKM2.

**Table 2:** Expression of p-EGFR and PKM2 in lung tumors and paired, normal, tumor-adjacent lung tissue.

				**–**	**+**	**++**	**+++**
Lung cancer (*n* = 35)			p-EGR	15	12	3	5
Well-differentiated lung carcinoma (*n* = 11)	Moderately differentiated lung carcinoma (*n* = 13)	Poorly differentiated carcinoma(*n* = 11)					
			PKM2	8	13	2	12
Paired normal tumor-adjacent lung tissues (*n* = 35)			p-EGFR	32	3	0	0
			PKM2	28	1	3	3

*Expression of p-EGFR and PKM2 in lung cancer tissues. Statistical data of immunohistochemical staining of p-EGFR and PKM2 in 35 lung-tumor samples and matched, adjacent tissue. “–,” “+”, “++,” and “+++” indicate increments of positive staining*.

## Discussion

Arsenic induces skin, lung, and other human cancers. Recent studies into the carcinogenic properties of arsenic have focused on how exposure affects genetic changes, the generation of reactive oxygen species, epigenetic alterations, and expression of miRNAs (33). Our finding that arsenic activates EGFR in lung cancer is supported by reported observations of EGFR activation by either 100 μM arsenite or 800 μM arsenate in another keratinocyte type (19, 34).

Reportedly, arsenic induces EGFR phosphorylation nearly as efficiently as engagement of EGFR with its ligand, EGF. Furthermore, EGFR is not degraded in response to activation by arsenic (19). Our data showed arsenic exposure caused HB-EGF to stimulate EGFR phosphorylation (p-EGFR) at Tyr 1068 and confirmed HB-EGF was highly expressed in As-T cells and most tumor tissues samples of 35 lung cancer cases. The latter observation consist with study by Yotsumoto and Hsieh that HB-EGF is the predominant EGFR ligand in lung cancer cells and EGFR mutation and that HB-EGF expression predicts poor survival in patients with primary lung tumors (35, 36).

HB-EGF is reported to be a promising therapeutic target for lung cancer and ovarian cancer (37). In support of this idea, we find it plays an important role in many aspects of arsenic-induced lung cancer: HB-EGF induced As-T and A549 cell proliferation, colony formation, migration, and tumor formation capability.

EGFR activation induces translocation of PKM2 into the nucleus, where PKM2 acts both as a protein kinase and a transcriptional coactivator for hypoxia-inducible factor alpha (HIF-1α) in HeLa cervical carcinoma cells (21, 38). PKM2 is known to promote cell migration and inhibits autophagy by mediating PI3K/AKT activation in gastric cancer or metastasis by recruiting myeloid-derived suppressor cells; and PKM2 expression is a marker of poor prognosis in hepatocellular carcinoma (39, 40). Our studies demonstrated that, in As-T cells, PKM2 upregulated HIF-1α at the transcriptional level. Furthermore, we found HB-EGF inhibitors could downregulate HIF-1α at the transcriptional level, via ERK. More meaningfully, PKM2 also induced As-T cell proliferation and migration. Our data obtained from samples showed PKM2 was overexpressed in most cases of 35 lung cancer compared to expression in matched, adjacent, normal tissue. These results indicated that PKM2 played oncogenic role in As-T cells.

Kanematsu et al. reported EGFR phosphorylation but not overexpression was correlated with poor prognosis of non-small cell lung cancer patients (41). In agreement, our results detected significantly higher expression of p-EGFR in 20/35 cases of lung cancer (as compared to matched, adjacent, normal tissue); and also demonstrated a positive correlation between HB-EGF and p-EGFR, suggesting HB-EGF may be the main trigger for EGFR phosphorylation in lung cancer.

In conclusion, HB-EGF played an important role in As-T cells, and activated EGFR and downstream signal pathways in lung cancer. HB-EGF may also be a rational target for lung cancer treatment, and CRM197 might be expected to improve prognosis in patients with NSCLC.

## Data Availability Statement

The datasets generated for this study are available on request to the corresponding author.

## Ethics Statement

The animal study was reviewed and approved by the Ethics Committee of Nanjing Medical University.

## Author Contributions

LW and Y-FL carried out the samples analysis and performed the experiments. C-SW, Y-XX, YQ-Z, Y-CQ, and W-TL conducted the cell function experiments. MW and B-HJ wrote the manuscript. All authors contributed to the article and approved the submitted version.

## Conflict of Interest

The authors declare that the research was conducted in the absence of any commercial or financial relationships that could be construed as a potential conflict of interest.
